# Anticoagulation Following Surgery for Glioblastoma: Case Report and Review of the Literature

**DOI:** 10.1055/a-2952-7250

**Published:** 2026-09-17

**Authors:** Zade Akras, David I. Nawabi, Noah L. A. Nawabi, Adam Glaser, Lennard Spanehl, Florian A. Gessler, Pablo Valdes, Pierpaolo Peruzzi, Omar Arnaout, E. Antonio Chiocca, Timothy R. Smith, Joshua D. Bernstock

**Affiliations:** 1 Department of Neurosurgery 1861 Brigham and Women’s HospitalBostonMassachusettsUnited States; 2 1811 Harvard Medical SchoolBostonMassachusettsUnited States; 3 College of Medicine 2345 Medical University of South CarolinaCharlestonSouth CarolinaUnited States; 4 Department of NeurosurgeryUniversity Medicine RostockRostockGermany; 5 Department of NeurosurgeryUniversity of Texas Medical BranchGalvestonTexasUnited States

**Keywords:** glioblastoma, anticoagulation, intracranial hemorrhage, venous thromboembolism, inferior vena cava filter

## Abstract

Patients with glioblastoma (GBM) have a high risk of venous thromboembolism (VTE), particularly after surgery, but a competing high risk of intracranial hemorrhage complicates the need for anticoagulation. We present a case of a 70-year-old male on dabigatran with a history of VTE who underwent GBM resection. A preoperative inferior vena cava (IVC) filter was placed to allow for temporary postoperative holding of chemical anticoagulation. Despite this, he developed progressive, life-threatening thromboembolism, including bilateral deep vein thromboses, a pulmonary embolism, and ultimately extensive thrombosis of the IVC extending into the right atrium, causing obstructive shock. This progression occurred despite the resumption of anticoagulation and necessitated a high-risk mechanical thrombectomy, which was complicated by cardiac arrest secondary to right atrial perforation. The patient ultimately survived. Given the questions raised by this case, we review the literature on the use of anticoagulation following surgery for GBM. We highlight the distinct risk profiles associated with prophylactic and therapeutic anticoagulation and suggest early thromboprophylaxis when not contraindicated.

## Background

Venous thromboembolism (VTE), including deep vein thrombosis (DVT) and pulmonary embolism (PE), is a major cause of cancer-related morbidity and mortality. 1 Patients with glioma are at particularly high risk of VTE, with a 30% incidence in glioblastoma (GBM) patients over the course of the disease. Concerns regarding intracranial hemorrhage (ICH) and the uncertain relative impact of VTE prophylaxis versus ICH on overall survival complicate decisions regarding thromboprophylaxis in this population. 2 3 4 5 6 This tradeoff is especially relevant in the postoperative period following craniotomy for tumor resection, during which patients are at increased risk for both types of events. 7 We report the case of a patient receiving dabigatran for a remote history of VTE who presented to our center for resection of a GBM. The case prompted extensive multidisciplinary discussion regarding optimal perioperative VTE prophylaxis and anticoagulation management. In light of the clinical questions raised, we also review the existing literature on anticoagulation use following surgical resection of GBM.

## Case Presentation

**Fig. 1:**
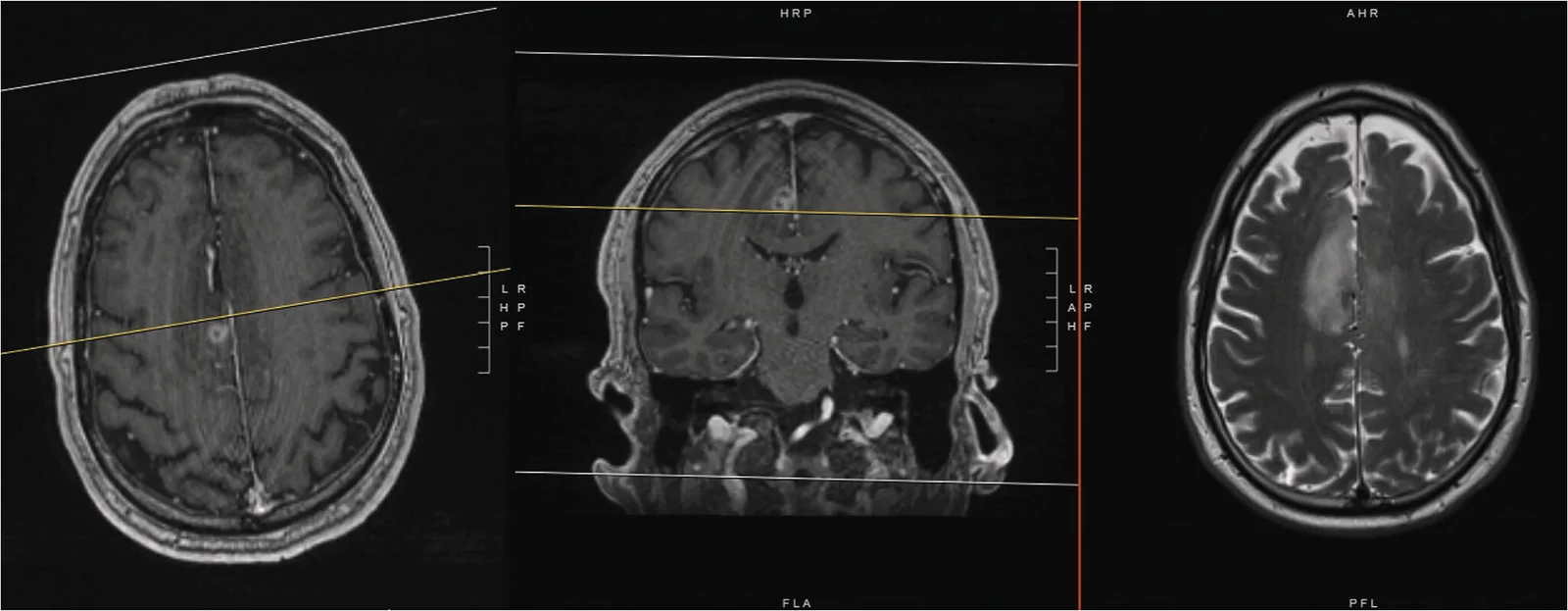
Preoperative MRI showing right frontal glioblastoma (GBM).

A 70-year-old male with a remote history of DVT and PE (on dabigatran), hypertension, hyperlipidemia, and benign prostatic hyperplasia presented to an outside hospital with new focal seizures. Magnetic resonance imaging (MRI) revealed a 10-mm enhancing paramedian right frontal GBM ( Fig. 1 ), and he was transferred to our center for further management. The patient previously had a negative workup for hypercoagulability syndromes including factor V Leiden, prothrombin gene mutation, antithrombin III deficiency, protein C/S deficiency, and anticardiolipin and lupus anticoagulant antibodies. There was extensive discussion with the vascular medicine team regarding his thrombotic and bleeding risk management perioperatively. The decision was made to place an inferior vena cava (IVC) filter prior to undergoing a right craniotomy for tumor resection and hold anticoagulation postoperatively, with plans to resume direct oral anticoagulation (DOAC) at postoperative day (POD)10 and retrieve the filter at that time. Dabigatran was held 6 days prior to surgery, and he was bridged on a heparin drip until 8 hours prior to surgery. Pneumatic compression devices were applied intraoperatively as is routine at our center.

**Fig. 2:**
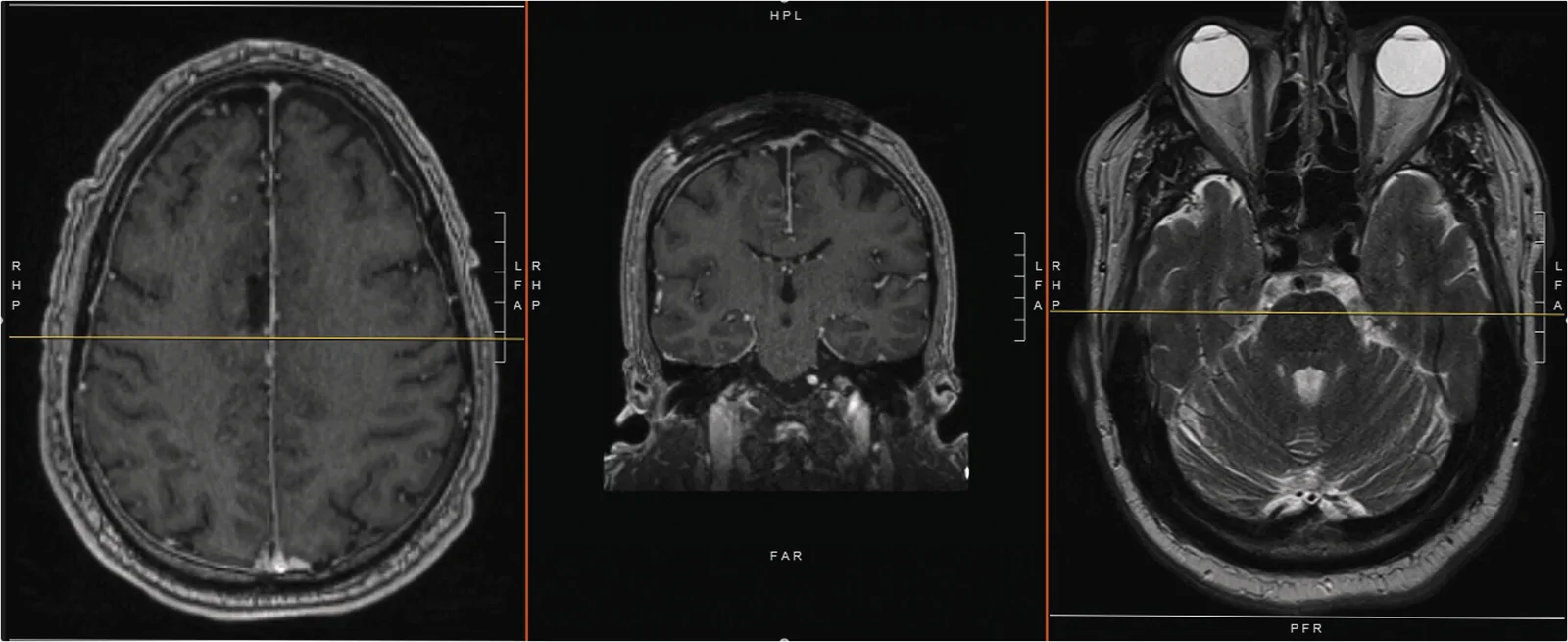
Postoperative MRI showing gross total resection.

Gross total resection was achieved ( Fig. 2 ). The procedure time was 193 minutes, and the total in-room time was 307 minutes. The patient tolerated the procedure well and was transferred to the floor on POD1 in stable condition. On POD2, he was started on subcutaneous heparin. Lower extremity ultrasonography showed bilateral gastrocnemius and right soleal DVTs, but the extent of thrombus was not deemed to necessitate any changes to his anticoagulation plan. He continued to recover well until POD5, when a rapid response was called after the patient had a witnessed fall. He was found to be hypotensive with a systolic pressure in the 70s and an electrocardiogram showing sinus tachycardia. Computed tomography (CT) of the chest and neck revealed a right lobe subsegmental PE. In the ensuing days, he developed a transient acute kidney injury, believed to be prerenal secondary to postobstructive diuresis. Renal artery ultrasonography was performed, which did not show thrombosis. On POD8, he was started on a heparin drip, and his home dabigatran was restarted the next day after a CT of the head confirmed no acute hemorrhage.

He continued to recover well until POD14, when he had worsening lower extremity swelling. Ultrasonography was repeated, this time showing right and left common femoral vein, femoral vein, popliteal, and gastrocnemius DVTs with acute features. The vascular medicine service was re-engaged given the progression of his DVTs despite being on dabigatran. Due to the lack of clarity regarding whether he had new DVTs or had worsening of the existing DVTs prior to being on dabigatran, it was deemed best to switch the anticoagulation strategy. Half-dose low-molecular-weight heparin (LMWH) was started with plans to escalate to a full 1 mg/kg dosage after a CT of the head 2 days later.

**Fig. 3:**
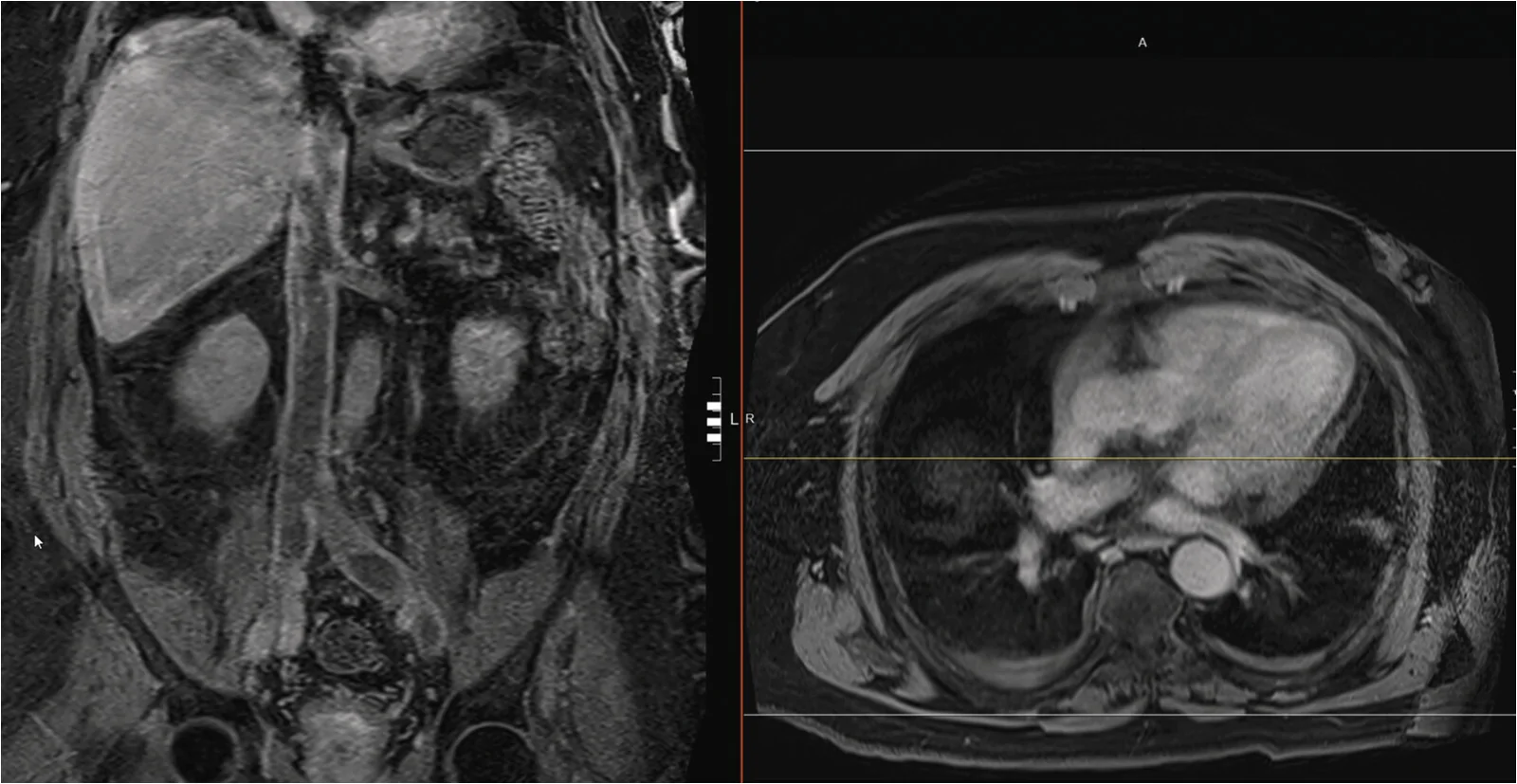
Abdominal MRI showing extent of thrombus.

On POD15, the patient had another presyncopal episode with systolic pressure in the 80s. He was found to have acute kidney injury and a hepatocellular pattern of liver injury. Abdominal ultrasonography was performed on POD16, which was concerning for an intrahepatic IVC thrombosis. Transthoracic echocardiography showed a small pericardial effusion and normal right and left ventricular systolic function. MRI demonstrated extensive thrombosis involving the IVC extending to the right atrium, hepatic veins, renal veins, iliac veins, and left common femoral vein ( Fig. 3 ). An urgent CT of the head was also performed to better inform a risk–benefit decision regarding intracranial bleeding, which showed decreased blood products from prior hemorrhage and no acute hemorrhage. Following consultation with the vascular medicine and gastroenterology teams, it was agreed that a therapeutic heparin drip would be the most appropriate course of action. However, following consultation with the pharmacy team, there was concern that treating with heparin or additional LMWH would result in his anticoagulation becoming supratherapeutic due to the patient’s impaired renal function. An anti-Xa assay was thus collected 6 hours after the last LMWH dose prior to starting therapeutic heparin.

**Fig. 4:**
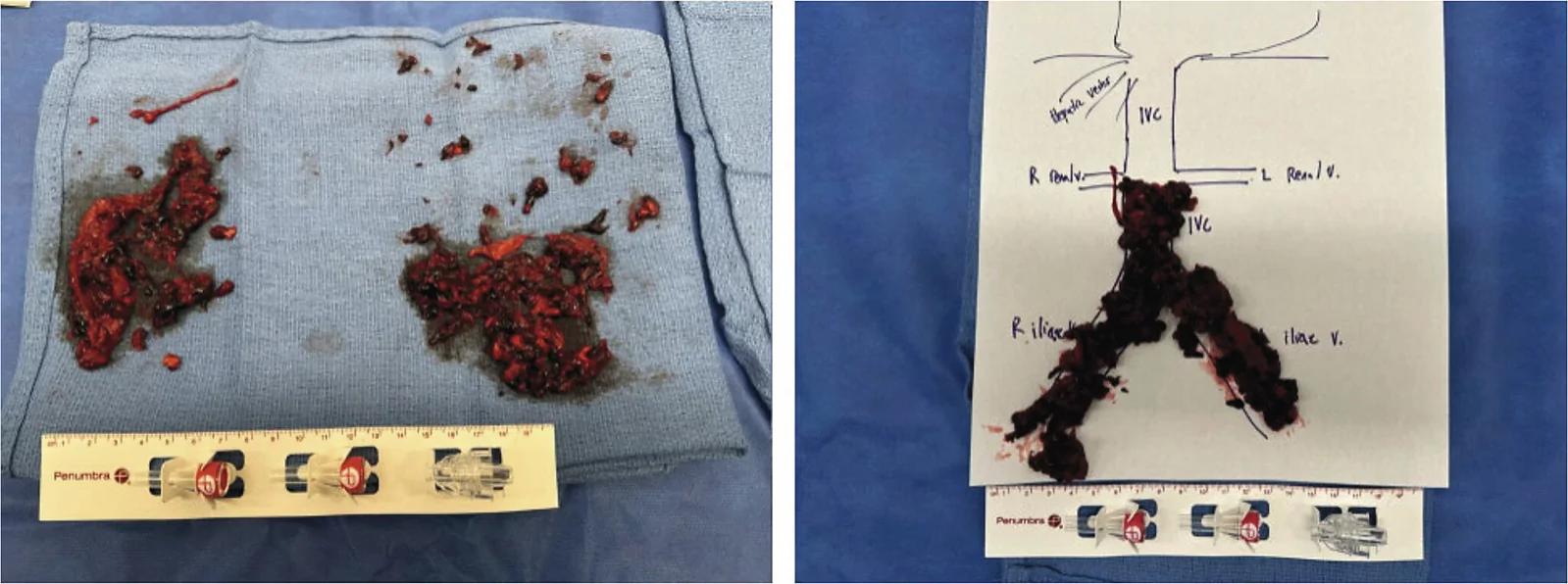
Clot removed by mechanical thrombectomy.

The interventional radiology service was consulted, who agreed that mechanical thrombectomy and IVC filter retrieval were warranted despite being high-risk. Preprocedure imaging revealed that the IVC filter had migrated from its original infrarenal position to the level of the IVC/right atrial junction. Following near-complete removal of the thrombus, removal of the IVC filter was complicated by intraoperative pulseless electrical activity. Multiple rounds of chest compressions were performed, and return of spontaneous circulation was achieved after approximately 8 minutes, but the patient continued to have hemodynamic instability. Intraoperative transesophageal echocardiography showed severe underfilling of the left ventricle, periodic severe pericardial effusion, and a massive left pleural effusion, suspicious for cardiac injury and drainage into the left pleural space. The thoracic surgery team placed a left-sided chest tube, which drained 4 liters of blood. Given the high suspicion for a cardiac or major vessel injury, he was taken by the cardiac surgery team for a chest exploration, where he was found to have a perforation of the right atrium and IVC. An exploratory laparotomy was also performed by the acute care surgery team who did not find any extension of the IVC injury below the diaphragm. After delayed chest closure, he returned to interventional radiology who successfully completed thrombectomy and removal of the IVC filter, and he was extubated on POD21 from the index operation ( Fig. 4 ). He was discharged to an inpatient rehabilitation center on POD38, with discharge examination notable for intact extraocular movement, symmetric face with midline tongue, grossly intact sensation, impaired gait and balance, and ⅘ strength throughout, and was discharged home from rehab 3 weeks later.

## Discussion

**Fig. 5:**
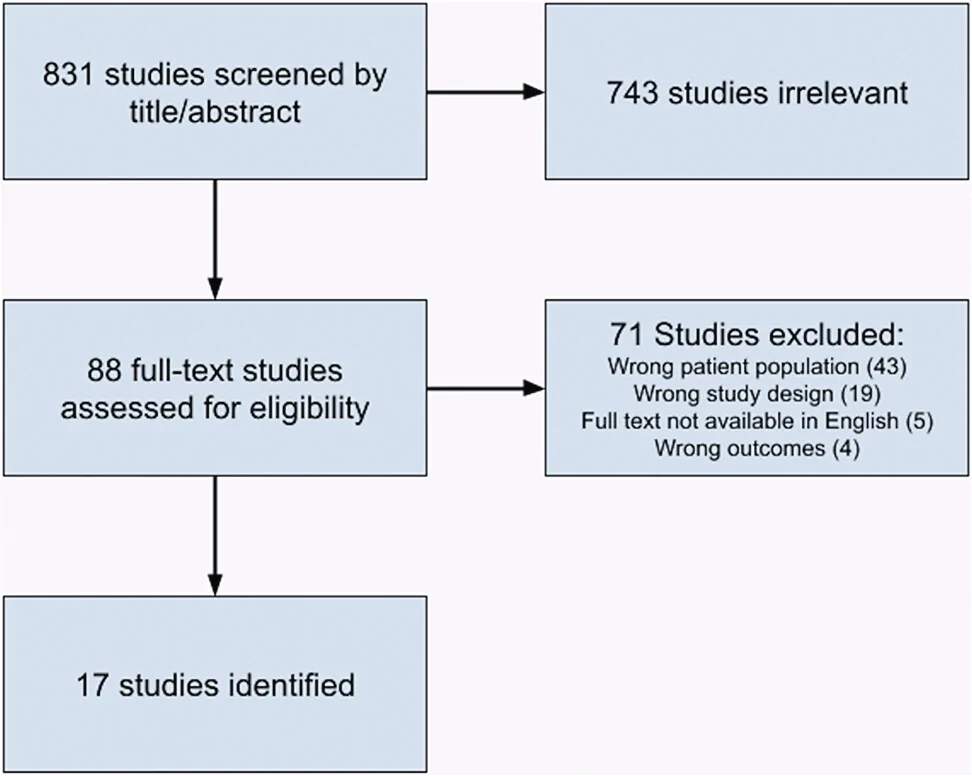
PRISMA flow diagram of literature review **.** On October 10, 2025, the following search string was used: (glioblastoma OR GBM OR glioma) AND (anticoagulant OR anticoagulation OR heparin OR “low molecular weight heparin” OR LMWH OR enoxaparin OR dalteparin OR tinzaparin OR nadroparin OR bemiparin OR certoparin OR warfarin OR DOAC OR “direct oral anticoagulant” OR apixaban OR rivaroxaban OR edoxaban OR betrixaban OR dabigatran).

Our case illustrates the challenges in anticoagulation management in patients with GBM, who are affected by a spectrum of patient-, tumor-, and treatment-associated variables that increase VTE risk. 8 Motivated by this case, we performed a MEDLINE search for papers on anticoagulation in patients following surgical resection of GBM ( Fig. 5 ). Papers were included if they reported the risk of VTE and/or ICH in the postoperative period following surgical resection of GBM, under no anticoagulation, prophylactic anticoagulation, or therapeutic anticoagulation following VTE. Exclusion criteria included: case reports, reviews, or meta-analyses; studies including all patients undergoing craniotomy without reporting specific subgroup data for GBM; studies reporting interventions and outcomes not specified to surgical patients, e.g., focused on ambulatory GBM patients or patients admitted for radiation or chemotherapy; and studies describing characteristics of patients experiencing VTE/ICH postoperatively without reporting the incidences of these events as a percentage of all eligible patients. Sixteen studies described the incidences of VTE and/or ICH in surgical GBM patients ( Table 1 ). Seven studies described the risk of ICH and/or recurrent VTE in surgical GBM patients who experienced VTE ( Table 2 ). Our review identified significant variability in anticoagulation protocols following GBM resection but demonstrated consistent patterns in VTE risk among these patient populations.

**Table 1:** Studies reporting incidences of venous thromboembolism (VTE) and/or intracranial hemorrhage (ICH) following surgery for glioblastoma (perioperative VTE/ICH is defined as occurring within 30 days of surgery [35 days in Eisele 35 ], or explicitly described as postoperative or perioperative in the study; total is defined according to the last study follow-up).

Study (Author, Year)	Study design	Prophylactic AC	Number of patients in arm	Perioperative VTE: *N* (%)	Perioperative ICH: *N* (%)	Total VTE: *N* (%)	Total ICH: *N* (%)	Notes
Brandes, 1997 33	Prospective	Subcutaneous calcium heparin 5,000 IU TID for 20 days following surgery	61	0 (0%)	0 (0%)	19 (31%)	0 (0%)	
Dubinski, 2022 14	Retrospective	20 mg Clexane (LMWH) at 12 h → 40 mg on POD1	584	46 (8%)				
Ebeling, 2018 34	Retrospective	LMWH initiated 1–3 days after surgery	153	12 (8%)				
Edwin, 2016 6	Retrospective	Not specified	450	55 (12%)		145 (32%)		
Eisele, 2022 35	Retrospective	82% of patients had unspecified preoperative prophylaxis	414	27 (7%)		65 (16%)		
Kapteijn, 2023 36	Retrospective	Not specified	324			25 (8%)	34 (10%)	Median time to VTE of 3 months after diagnosis
Kaptein, 2022 7	Retrospective	Nadroparin (LMWH) 2,850 IU once daily, or 5,700 IU once daily when body weight >100 kg or presence of ≥1 additional VTE risk factor	967	14 (1%)	57 (6%)	101 (10%)	118 (12%)	Frequency of perioperative ICH estimated from frequencies of perioperative bleed and proportion of bleeds that were ICH
Khoury, 2016 9	Retrospective	Not specified	523	51 (10%)		173 (33%)	17 (3%)	
Pan, 2009 37	Retrospective	None (except for 3 patients below)	146	11 (8%)		41 (28%)	1 (1%)	
		Aspirin for 2 months	2				0 (0%)	
		Warfarin for 8 months for atrial fibrillation	1				0 (0%)	
Sawaya, 1992 38	Prospective	None	15	9 (60%)				VTE detected prospectively by postoperative ^125^ I-labeled fibrinogen leg scans
Senders, 2018 23	Retrospective	Dalteparin (LMWH) until discharge (0–7 days) or until 21 days	264	6 (2%)	9 (3%)	20 (8%)		
Simanek, 2007 39	Prospective	LMWH during first 10 postoperative days: 40 mg enoxaparin, 2,500 IU dalteparin, or 5,000 IU dalteparin daily	51			12 (24%)		Cumulative probability of VTE 21% after 3 months
Thomas, 2022 21	Prospective	Apixaban (DOAC) 2.5 mg BID beginning 2–21 days after craniotomy and continued for 6 months as tolerated; 70% of patients received postoperative LMWH	9	0 (0%)	0 (0%)	0 (0%)	0 (0%)	
Wardenbach, 2025 13	Prospective	Certoparin (LMWH)	304			8 (3%)		Outcomes measured within 3 months post-surgery
		Enoxaparin (LMWH)	163			14 (9%)		
		Enoxaparin (LMWH) + PCD	228			6 (3%)		
Watanabe, 2019 11		None	110			32 (29%)		Patients with HGG were prospectively screened with serial D-dimer levels; majority of VTE occurred within 14 days of surgery, but specific timing breakdown not reported for GBM subgroup
Wilhelmy, 2023 12	Retrospective	LMWH initiated on POD1 after head imaging, or 2–5 days after stabilization of postoperative ICH	222	8 (4%)	9 (4%)			Only 1 patient developed ICH after initiation of prophylactic AC

Abbreviations: AC, anticoagulation; DOAC, direct oral anticoagulation; HGG, high-grade glioma; LMWH, low-molecular-weight heparin; PCD, pneumatic compression device; POD, postoperative day.

**Table 2:** Studies reporting outcomes following occurrence of venous thromboembolism (VTE).

Study (Author, Year)	Study design	Therapeutic AC	Number of patients in arm	ICH: *N* (%)	Recurrent VTE: *N* (%)
Dubinski, 2022 14	Retrospective	LMWH	32	2 (6%)	0 (0%)
		LMWH → DOAC on POD14	14	1 (7%)	0 (0%)
Edwin, 2016 6	Retrospective	Heparin/LMWH: 54 (72%); Warfarin: 20 (27%); DOAC: 1 (1%)	75		16 (21%)
Eisele, 2022 35	Retrospective	LMWH: 29 (48%) Warfarin: 26 (43%) DOAC: 4 (7%) Heparin: 2 (3%)	61	7 (11%)	2 (3%)
Khoury, 2016 9	Retrospective	None	76	2 (3%)	
		LMWH	69	11 (16%)	
		Warfarin	26	3 (12%)	
		Heparin	2	1 (50%)	
Pan, 2009 37	Retrospective	LMWH +/− warfarin	25	2 (8%)	3 (12%)
		No AC	14	1 (7%)	4 (29%)
		Antiplatelet therapy	2	0 (0%)	0 (0%)
Schmidt, 2002 40	Prospective	LMWH 175 IU/kg for 10 days, followed by 100 IU/kg for 3 months or 75 IU/kg if thrombocytopenia	9	0 (0%)	2 (22%)
Wilhelmy, 2023 12	Prospective	Not specified	8	3 (38%)	

Abbreviations: AC, anticoagulation; DOAC, direct oral anticoagulation; ICH, intracranial hemorrhage; LMWH, low-molecular-weight heparin; POD, postoperative day.

VTE occurs in more than 30% of patients with GBM. 2 6 9 While only 3% of patients undergoing craniotomy for any brain tumor have perioperative VTE, high-grade glioma is independently associated with increased VTE risk. 10 The postoperative period is particularly high-risk, with 20% of patients having postoperative VTE 4 11 and estimates ranging from 40% of VTEs occurring within 30 days of surgery up to 80% occurring within 7 days of surgery. 6 11 Patients with VTEs have worse in-hospital 12 and overall 7 mortality.

When GBM patients are anticoagulated with LMWH or DOACs postoperatively, the incidence of VTE drops to 2 to 9% within 3 months of surgery and to 10% over a median observation time of 15 months. 7 13 14 However, anticoagulation raises concerns regarding ICH, a devastating complication of both craniotomy and intracranial malignancy, up to half of which occur in the first week after surgery. 7 ICH is associated with a 32% in-hospital and 45% 1-year mortality 15 and is the leading cause of reoperation following craniotomy for primary malignant brain tumors. 16 These risks are amplified in patients on anticoagulation, who experience worse outcomes following ICH compared with those not on anticoagulation. 15 17

Although prophylactic anticoagulation raises concern for ICH, physicians must also weigh the potential need for therapeutic-dose anticoagulation or life-saving thrombolytic therapy if VTE does occur. Our review of the literature revealed higher incidences of ICH in GBM patients receiving therapeutic anticoagulation following a VTE. Across craniotomies for brain tumors, 4% of patients with VTE treated with unfractionated or LMWH develop ICH, 10 whereas this rate increases to as high as 17% in GBM patients. 9 Two meta-analyses similarly found that patients with glioma receiving therapeutic anticoagulation for VTE have a 3.6- to 3.8-fold increased risk of ICH. 18 19 In high-grade glioma, a time-adjusted model found patients have a 13-fold increased risk of ICH while receiving therapeutic enoxaparin following a VTE. 17 Another study found major bleeds occurred in 50% of surgical GBM patients who developed PE. 12 A final study found that while only 13% of patients with ICH were anticoagulated at the time of their bleed, 75% of these had started anticoagulation secondary to an incident VTE. 7 Catheter-directed therapies may be considered in select high-risk patients; however, these interventions also carry significant risk, as illustrated by our patient. 20

In contrast to therapeutic anticoagulation, prophylactic anticoagulation presents a more favorable risk profile. Early initiation of prophylactic anticoagulation with heparin or enoxaparin, on POD1, has been shown to decrease incidence of VTE without associated hemorrhagic complications. 4 12 DOACs have also shown safety profiles comparable or even preferable to LMWH in retrospective and small prospective studies. 14 21 22 Prolonged prophylaxis is not without risk, however. A cohort of patients undergoing resection for high-grade glioma demonstrated that LMWH prophylaxis for 21 days following surgery is not associated with reduced VTE within 21 days but is associated with a significant increase in ICH. 23 A randomized placebo-controlled trial found long-term LMWH use for 6 months following surgery does reduce VTE incidence compared with placebo but also causes increased ICH, with similar 12-month mortality in the two groups. 24 Intermittent pneumatic compression is also a low-risk option that may further reduce VTE risk even in patients already receiving chemoprophylaxis. 25

Decisions regarding anticoagulation should therefore be individualized, taking into account each patient’s clinical context and personal risk factors. 26 Adjuvant oncologic therapies—including chemotherapy, radiation, and immunotherapy—may further increase the risk of VTE. 7 27 Our patient was receiving dabigatran for a prior history of VTE. Although his previous hypercoagulability workup was negative, a history of VTE remains the strongest predictor of thromboembolic events in the postoperative period. 4 28 Accordingly, the clinical question was not whether to initiate anticoagulation, but rather when it could be safely resumed. Placement of an IVC filter was considered an attractive bridging strategy in the setting of heightened concern for postoperative hemorrhage following craniotomy and GBM resection, with a plan to resume dabigatran on POD10. However, this strategy proved inadequate in preventing PE, possibly due to migration of the filter. Fatal PE following GBM resection despite placement of an IVC filter has been previously reported, 29 and 33% of GBM patients treated with IVC filters develop recurrent VTE. 6 Data in patients with cancer suggest that IVC filters confer uncertain benefit while increasing the risk of filter-related DVT and IVC thrombosis, which may have contributed to the outcome in our patient. 5 30 Finally, while routine DVT screening is unlikely to confer a benefit to neurosurgical patients already receiving standard-of-care thromboprophylaxis, it could be considered in select patients. 31 32 Our patient did have DVTs identified on lower extremity ultrasonography on POD2, and while they were not deemed to necessitate adjustment of his postoperative anticoagulation plan, further studies are warranted to elucidate the appropriate clinical response to positive DVT screening.

## Conclusion

Our case demonstrates a major morbidity caused by VTE following surgery for GBM. While ICH can be similarly devastating, the risk–benefit tradeoff requires understanding the distinction between prophylactic and therapeutic anticoagulation. Prophylactic anticoagulation reduces VTE risk and is safe in the immediate postoperative period, when VTE risk is at its highest. Delays in thromboprophylaxis risk direct complications from VTE, as well as from future major bleeds or other complications if therapeutic-dose anticoagulation or catheter-directed interventions are required. This case highlights the need for a systematic review and meta-analysis to identify the evidence-based anticoagulation strategies for patients undergoing surgery for GBM.

## Data Availability

There were no novel datasets generated for this study.

## Declaration of GenAI Use

Gemini 3 Pro was used to proofread the final manuscript.
